# Evidence for preserved insulin responsiveness in the aging rat brain

**DOI:** 10.1007/s11357-022-00618-z

**Published:** 2022-07-08

**Authors:** Matthew G. Engel, Jeremy Smith, Kai Mao, Gabriela Farias Quipildor, Min-Hui Cui, Maria Gulinello, Craig A. Branch, Samuel E. Gandy, Derek M. Huffman

**Affiliations:** 1 Department of Molecular Pharmacology, Albert Einstein College of Medicine, Bronx, NY 10461 USA; 2 Institute for Aging Research, Albert Einstein College of Medicine, 1300 Morris Park Ave, Golding Building Room 201, BronxBronx, NY 10461 USA; 3 Department of Radiology and Imaging Sciences, Emory University School of Medicine, Atlanta, GA USA; 4 Department of Medicine, Albert Einstein College of Medicine, Bronx, NY 10461 USA; 5 Department of Radiology, Albert Einstein College of Medicine, Bronx, NY 10461 USA; 6 Dominick S. Purpura Department of Neuroscience, Behavioral Core Facility, Albert Einstein College of Medicine, Bronx, NY USA; 7 Department of Neurology and the Mount Sinai Center for Cognitive Health, Icahn School of Medicine at Mount Sinai, New York, NY 10029 USA; 8 Department of Psychiatry and the Mount Sinai Alzheimer’s Disease Research Center, Icahn School of Medicine at Mount Sinai, New York, NY 10029 USA

**Keywords:** Insulin, Responsiveness, Aging

## Abstract

Insulin appears to exert salutary effects in the central nervous system (CNS). Thus, brain insulin resistance has been proposed to play a role in brain aging and dementia but is conceptually complex and unlikely to fit classic definitions established in peripheral tissues. Thus, we sought to characterize brain insulin responsiveness in young (4–5 months) and old (24 months) FBN male rats using a diverse set of assays to determine the extent to which insulin effects in the CNS are impaired with age. When performing hyperinsulinemic-euglycemic clamps in rats, intracerebroventricular (ICV) infusion of insulin in old animals improved peripheral insulin sensitivity by nearly two-fold over old controls and comparable to young rats, suggesting preservation of this insulin-triggered response in aging per se (*p* < 0.05). We next used an imaging-based approach by comparing ICV vehicle versus insulin and performed resting state functional magnetic resonance imaging (rs-fMRI) to evaluate age- and insulin-related changes in network connectivity within the default mode network. In aging, lower connectivity between the mesial temporal (MT) region and other areas, as well as reduced MT signal complexity, was observed in old rats, which correlated with greater cognitive deficits in old. Despite these stark differences, ICV insulin failed to elicit any significant alteration to the BOLD signal in young rats, while a significant deviation of the BOLD signal was observed in older animals, characterized by augmentation in regions of the septal nucleus and hypothalamus, and reduction in thalamus and nucleus accumbens. In contrast, ex vivo stimulation of hippocampus with 10 nM insulin revealed increased Akt activation in young (*p* < 0.05), but not old rats. Despite similar circulating levels of insulin and IGF-1, cerebrospinal fluid concentrations of these ligands were reduced with age. Thus, these data highlight the complexity of capturing brain insulin action and demonstrate preserved or heightened brain responses to insulin with age, despite dampened canonical signaling, thereby suggesting impaired CNS input of these ligands may be a feature of reduced brain insulin action, providing further rationale for CNS replacement strategies.

## Introduction

Insulin and related peptides, such as insulin like growth factor-1 (IGF-1), contribute to early neurodevelopment as well as modulation of synaptic plasticity, neuronal survival, myelination, repair, growth, and proliferation in the adult brain [1]. In the hippocampus, insulin can reduce threshold frequency of activation for long-term potentiation (LTP) [2], promote neurogenesis, and play a key role in maintenance of neural stem cell pools by binding to insulin receptors (InsRs) [3]. Contrary to the historical characterization of the brain as insulin-independent (relying mainly on GLUT1 and GLUT3 transporters for glucose uptake) [4], mounting evidence supports a role for insulin signaling in the regulation of PI3K-dependent GLUT4 translocation in neuronal glucose utilization [5, 6], hypothalamic glucose sensing [7], and hippocampal spatial memory formation [8]. Moreover, the ability of central insulin to regulate peripheral metabolism has been well established by our group and others [9, 10].

While insulin/IGF-1 signaling play essential roles in central nervous system (CNS) development and homeostasis, its role in whole organismal and CNS aging have proven to be more controversial and potentially context-dependent. On one hand, a spontaneous or engineered reduction in insulin signaling can extend lifespan in model organisms [11, 12], yet peripheral insulin resistance in rodents and humans, as classically defined by an impaired ability of insulin to regulate glucose, is a salient feature of visceral obesity, type 2 diabetes (T2D), and aging [11]. Insulin resistance can also transiently serve as a protective response against excess nutrients in cells and tissues and redirect energy storage to adipose tissue [13]. The notion that insulin resistance may also be a manifestation of the aged and/or diseased CNS, either as a cause or consequence, has also been an area of intense investigation and debate, though there is no consensus as to how to best define insulin resistance in the brain and whether its existence can be established beyond an attenuation in canonical signaling [14].

Genetic models of low insulin/IGF-1 signaling in the CNS have been shown to promote longevity in *Drosophila* [15]. Likewise, brain-specific *IRS2* knockout improves lifespan in mice [16], and a reduction in IGF-1 receptors (IGF-1Rs) protects against the neurotoxic effects of cerebral amyloidosis [17]. However, there is also evidence that dysregulation of insulin signaling in the mammalian CNS may contribute to age- and disease-related cognitive decline. Brain-specific reductions in InsRs (NIRKO mice) result in an impaired ability to regulate glucose homeostasis, brain cholesterol synthesis, and cognitive impairment [18, 19]. Indeed, hippocampal insulin resistance as well as combined InsR/IGF-1R inactivation by AAV-Cre targeting the hippocampus [20] impairs synaptic plasticity, learning, memory performance, and global cognitive function [2, 21, 22]. Likewise, early deficits in cerebral glucose metabolism precede the development of cognitive symptoms in insulin-resistant adults and in T2D [23], and systemic insulin resistance has been linked to Alzheimer’s disease (AD) and other dementias [14]. Post-mortem examination of insulin signaling in the hippocampi of AD patients revealed markedly attenuated activation of IRS-1 phosphorylation on Ser 616, a biomarker of insulin resistance [14], and correlated with Aβ plaque burden in these brains [24]. Alternatively, reductions in central insulin action with aging and various pathologic states have also been attributed to a loss of input from impaired insulin transport across the blood–brain barrier (BBB), rather than central insulin resistance per se [25], as saturable insulin transport into the CNS is negatively impacted by obesity [26], T2DM [27], AD [28], and aging [29–31]. Moreover, both hyperglycemia and induced hyperinsulinemia under euglycemic conditions in AD patients acutely enhance performance on memory tasks [32], suggesting a deficiency in glucose utilization by the AD brain that can be partially rescued. Such observations helped to establish the premise of intranasal insulin as a therapeutic strategy for mild cognitive impairment and AD [33].

In order to further explore the concept of impaired insulin action in the aged CNS, we evaluated the response of the aging rat brain to acute insulin exposure at the level of canonical signaling and functional in vivo readouts, including central regulation of glucose homeostasis via hyperinsulinemic-euglycemic clamps and resting-state functional magnetic resonance imaging (rs-fMRI) detection of low-frequency variance in blood oxygen-level dependent (BOLD) signals [34]. Intriguingly, the results of this study suggest that the CNS appears to retain exquisite responsiveness to insulin in aging with regard to distinct functional assays involving the brain, in spite of an apparent inability to trigger a detectable level of PI3K-Akt signaling in bulk tissue. Moreover, cerebrospinal fluid (CSF) levels of insulin and IGF-1 were lower in old animals, despite similar levels in the periphery, thereby collectively suggesting a role for reduced entry into the CNS, rather than resistance per se, in impaired CNS insulin action with age.

## Methods

### Animals and experiments

Young (4 months) and old (24 months) male Fisher × Brown Norway (FBN) F1 hybrid rats were obtained from NIA for all experiments, housed at 22 °C under 14:10 light–dark photoperiod, and given access to water and regular chow ad libitum, with exception of Experiment 4, where a purified diet was provided. For Experiment 1, a cohort of young (*n* = 10) and old (*n* = 11) animals were used for basic phenotypic assessment and collection of various tissues and plasma for hormonal assays. For experiment 2, hyperinsulinemic-euglycemic clamps were performed in a cohort of young (*n* = 8) and old (*n* = 35) rats, with the latter being assigned to one of three treatment groups. For Experiment 3, body weight, composition, and behavioral assessment was performed, and discrete brain regions were freshly isolated for qRT-PCR and Western blotting in a cohort of young (*n* = 13) and old (*n* = 11) rats. In Experiment 4, a cohort of young (*n* = 8) and old rats (*n* = 9) was provided a purified diet (D12450B) for 8–10 weeks prior to performing a series of fMRI imaging studies. For Experiment 5, a cohort of young (*n* = 10) and old (*n* = 10) rats was used to obtain fresh brain tissue for the ex vivo insulin stimulation study. For Experiment 6, cerebrospinal fluid (CSF) collection, as well as blood and tissue collection, was performed under sedation in a cohort of young (*n* = 10) and old (*n* = 10) rats. All clamp, imaging and specimen collections were performed following a minimum 3–4 h fast. Experiments were approved by the Albert Einstein College of Medicine Institutional Animal Care and Use Committee.

### Surgical procedures

All surgical procedures were conducted under 2% Isoflurane. Stereotactic intracerebroventricular (ICV) placement of a steel-guide cannula for clamp studies or a similar non-metallic cannula for imaging studies (Plastics One, Roanoke, VA) reaching the 3rd ventricle was performed in rats (coordinates from bregma: + 0.2 mm A/P, − 9.0 mm D/V, 0.0 directly on the midsagittal suture) and the implant was secured in place with dental cement and animal treated with analgesic for 3 days as previously described [10]. Approximately 14 days later, animals assigned to clamp studies were sedated a second time for surgical placement of indwelling catheters into the right internal jugular vein and the left carotid artery as described [10, 35, 36]. Recovery was monitored until animals were within 3% of their pre-operative weight (typically 5–7 days) before conducting subsequent studies.

### Hyperinsulinemic-euglycemic clamp studies in rats

We performed hyperinsulinemic-euglycemic clamp studies with ICV infusion to evaluate central regulation of insulin sensitivity in aging FBN rats via insulin or IGF-1, respectively, as previously described [9, 10, 35]. In brief, all studies were 360 min in duration and consisted of a 120 min equilibration period, 120 min basal period, and a 120 min hyperinsulinemic clamp period. At the beginning of the study, animals were provided either a primed-continuous ICV infusion of aCSF, 1ug human IGF-I, delivered as a 0.3 µg bolus over 7.5 min, followed by a continuous infusion of 0.7 ug over 6 h (0.12 ug • h^−1^), or 30 uU insulin, provided first as a bolus of 7.5 uU over 7.5 min, then as a continuous infusion of 22.5 uU over 6 h (3.8uU • h^−1^) as described [10]. Following the basal period, a primed-continuous infusion of [3-^3^H]-glucose (20uCi bolus, 0.2uCi/min maintenance; NEN Life Science Products, Boston, MA) was given into the jugular vein and maintained throughout the remainder of the study. The hyperinsulinemic-euglycemic clamp was then initiated by peripheral administration of a primed-continuous infusion of regular insulin (3 mU • kg^−1^ • min^−1^), while somatostatin (1.5ug • kg^−1^ • min^−1^) was delivered to suppress endogenous insulin secretion. A 25% glucose solution was given and periodically adjusted to clamp the plasma glucose concentration at ~ 140–145 mg/dL. Serum samples for determination of [3-^3^H]-glucose and [3-^3^H]-glucose water specific activities (SAs) were obtained at 10-min intervals during the basal and clamp periods as described [9, 10].

### Behavioral and cognitive assessment

To confirm cognitive deficits in the NIA FBN F1 hybrid male cohort, young and old FBN F1 hybrid male rats were assessed for age-related changes in memory via object placement (OP), and startle and pre-pulse inhibition (PPI) invoked by acoustic startle reflex (ASR) in the Einstein Behavioral Core in a blinded fashion. OP is sensitive to deficits and amelioration in rat models of AD and aging [37–43]. To this end, rats were initially habituated to the open field, which consisted of an opaque arena (69 cm^2^). For testing, animals were placed in the arena containing two identical objects for 5 min and then returned to their original cages. After a retention interval of 90 min, animals were returned to the arena for a 6-min test interval, wherein one of the objects occupied a new position. Novel objects are typically preferred by normal animals [44]. Preference score was defined as the ratio of time exploring the novel object/total time exploring objects. Exploration was scored manually with a stopwatch and included sniffing, nosepokes, touching, and/or rearing on the object or orienting to and whisking and/or sniffing the object from a distance of 5 cm or less. Objects were positioned such that the intrinsic relationship between the objects was sufficient to identify the displaced object without using visual cues, albeit visual cues were present. All experiments were videotaped for confirmation and rigor. The pass/fail threshold for normal exploration was defined as 55% or greater preference for the novel object.

In rats, the ASR involves motor neuron activation by neurons in the caudal pontine reticular nucleus (PnC) after receipt of an initial auditory stimulus by cochlear neurons [45]. Startle and PPI invoked by ASR involve whole-body movement and eye blink in response to a sudden loud noise (110 dB). PPI is the reduction of this reflex by a lower intensity, non-startling noise preceding the startle stimulus by 40 ms, and ASR has been shown to decline in older humans [46]. To this end, young and aged rats were first placed in a startle chamber with specialized software (SD Instruments SR_Lab, San Diego, CA) and initially subjected to a 5–10-min acclimatization period consisting of background white noise. Animals then underwent a habituation period consisting of several (~ 10) 40 ms pulses at 100 dB at random time intervals in order to ensure a stable baseline and reduce variability. Next, the startle threshold was established prior to determining the startle magnitude with increasing amplitude. This consisted of 5 instances of 40 ms in duration for each dB level presented in random order with random intervals, and values were subsequently averaged for each animal. For the determination of PPI, 40-ms and 200-ms intervals, respectively, were performed with pre-pulse duration of 20 ms and target pulse 40-ms assessment and percent PPI were calculated as described [47].

### BOLD-fMRI and neural network analysis

For imaging studies, we performed a crossover design whereby young (*n* = 8) and old rats (*n* = 9) were assigned to undergo either the ICV CSF control or ICV insulin trial first, followed by a 1-week washout period and performance of the subsequent trial. To this end, animals were lightly sedated with isoflurane (1–1.5%) and resting-state-fMRI data acquisition was performed during the same time of day (1300–1600 h) over 60 min to provide approximately 900 data points per voxel. T_2_* acquisition, as well as low- (T_2_) and high-resolution (T_1_) anatomical acquisition, was obtained using a small-bore Varian 9.4 T MRI (Varian Medical Systems, Palo Alto, California) in the Einstein Gruss Magnetic Resonance Imaging Facility. Individual fMRI resting-state datasets were registered to a study-specific template (young rat selected at random) to serve as a common image, with the same pixel dimensions as the original, via rigid-body registration (i.e., the datasets were not resampled but shifted and rotated in space to match the common image). All further analyses at the individual and group level were performed in this common space. Images were then subjected to standard resting-state fMRI preprocessing steps, including correction for the timing of slice acquisition, motion correction, and lowpass filtering at 150 mHz, using the AFNI software suite (Cox, 1996). CSF and white matter signals, which represent confound (“nuisance”) signals of no interest, were estimated for each dataset using template CSF and white-matter masks. The first six principal components as well as the first derivative of the average signal were computed for the CSF and white matter tissues and regressed out of each pixel, along with the estimated effects of the six motion components (roll, pitch, yaw, and axial shifts) on pixel signal. A wavelet-based despiking routine developed by Patel and Bullmore [48, 49] was also applied as an additional denoising step. The first three volumes (~ 4.5 s) of each acquisition were discarded to allow data to reach T1-relaxation steady state. Analyses were conducted on the residual data in both the time domain and frequency (“spectral” or “Fourier”) domain. Frequency-domain data, which reflected the “spectral power” of pixel signals within each frequency band, was produced using the AFNI utility 3dPeriodogram.

For the seed-based correlation (SBC) analyses in the time domain, we registered the Waxholm atlas to the study template and extracted the average signal for each animal under four regions of interest as defined in the atlas: *mesial-temporal*, a combination of the hippocampus, perirhinal cortex, and entorhinal cortex; *hypothalamic*, which consisted of the hypothalamus and surrounding tissue; *thalamus*; and *cortex.* Correlation analyses were then performed for each animal and for each of these four “seed” signals using the AFNI utilities 3dDeconvolve and 3dREMLfit, which together compute a restricted maximum likelihood (REML) estimate for each “seed” region signal. Individual correlation results for each animal were combined and corrected for multiple comparisons (false-discovery-rate-corrected mixed effects meta-analysis) using the AFNI utility 3dMEMA. Pixels in these regions were also clustered based on the similarity of their signals, either in the time or frequency domain, via a “*K*-medoids” algorithm. This algorithm is very similar to *K*-means clustering, except that it leverages the $L_{1}$ norm or “Manhattan distance” as a metric, rather than the $L_{2}$ norm or “Euclidean distance” commonly employed in *K*-means. One young animal was excluded from the analysis due to a marked loss in body weight between scan 1 and scan 2 (> 20%), while one old animal died during the first scan (CSF treatment) and two other scans in aged animals were discontinued and related data were excluded from the analysis due to inadequate ventilation during the anesthesia and imaging episode.

### Ex vivo* insulin stimulation*

Following a 3 h fast, 4 months (young) and 24 months (old) male wild-type FBN rats (*n* = 10 per age group) were anaesthetized with 2.5% isoflurane and euthanized, and brains were minced to obtain 1 mm × 1 mm pieces from prefrontal cortex and hippocampus, respectively, and placed immediately in aCSF pre-warmed to 37 °C in 8-well plates. Following 10-0 min equilibration at 37 °C in 5% CO_2_, tissues were treated with aCSF (vehicle) or 10 nM insulin. Tissue pieces were then incubated at 37 °C in 5% CO_2_ for 2 min, washed with 1 × PBS, and then immediately dounce-homogenized on ice for extraction of protein with a modified RIPA buffer (150 mM NaCl, 50 mM Tris HCl, 0.25% deoxycholate, 5 mM EDTA, 100 µM PMSF, 1 mM orthovanadate, 5 mM sodium pyrophosphate, 1% Triton X-100, Roche protease inhibitor cocktail (1 × tablet per 10 mL), 100 mM NaF). Following a 15-min incubation on ice, samples were centrifuged and the supernatant containing solubilized protein was stored at − 80 °C for further analysis.

### Assays and analytical procedures

Serum glucose was monitored throughout the clamp via the glucose oxidase method with an Analox GM7 analyzer (Analox Inst., USA Inc, Lunenberg, MA). Endogenous insulin was measured by a rat/mouse ELISA (EMD Millipore, Inc, Billerica, MA) with rat insulin standards, and clamp insulin levels were measured by a human ELISA (ALPCO, Inc, Salem, NH) with human insulin standards. Endogenous IGF-1 and IGFBP-3 levels were measured in plasma via a validated “in-house” ELISA at the USC Aging Biomarker Service Core as described [50].

### Western blotting

The detection of protein levels was performed by Western blotting as described [10, 50, 51]. In brief, extracted protein concentration was determined using the BCA protein assay (Sigma, St. Louis, Mo). For electrophoresis, 20 µg of total protein was separated on Criterion TGX Stain-Free gels (4–20%, Bio-Rad). Stain-free gels were then imaged prior to transfer on a Bio-Rad Chemidoc MP Imaging System (Bio-Rad, Hercules, CA) to confirm equal protein loading. Gels were then wet transferred onto PVDF membranes at 100 V constant for 1 h, and equal transfer was confirmed by Ponceau S. Membranes were then blocked in 5% milk in TBST for 1 h at room temperature and then incubated overnight at 4 °C with primary antibodies from cell signaling against p-Akt^Thr308^ (1:1000; #13,038), total Akt (1:1000; #4691), p-p44/42MAPK^Thr202/Tyr204^ (1:1000; #9101) total p44/42 MAPK (1:1000; #4695), total IGF-1R (1:1000; #9750), and InsRβ (1:1000; #3025). Following a 1-h incubation with the appropriate secondary antibody. Clarity Western ECL Substrate (Bio-Rad, Hercules, CA) was applied to the membrane and bands were visualized using a Bio-Rad Chemidoc MP bioimager to first pixel saturation and densitometry was performed using Image Lab software (Bio-Rad, Hercules, CA).

### RNA isolation and expression

The total RNA from the frozen tissues was isolated using the TRIzol® Reagent per the manufacturer’s instructions (Life Technologies). First-strand complementary DNA (cDNA) was synthesized with random primers using Bio-Rad iScript cDNA Synthesis Kit. All qPCR reactions were carried out using Bio-Rad Sso Advanced SYBR Green mix on a Bio-Rad CFX384 qRT-PCR Machine. Target genes included *GRIN2B*, *TNFα*, *CDKN1A*, *IL1β*, *NFκB/p65*, and *CCL2*, and all data were normalized to *Actb*.

### Statistics

All data were analyzed by either Student’s tests for paired samples, independent sample *t*-tests based on the difference from baseline between two independent groups, and one-way ANOVA for GIR during the clamp and within the basal and clamp period for glucose production and disposal, respectively, and Post hoc analysis with Tukey adjustment was performed when appropriate. Object placement preference was analyzed by the chi-square test. Independent component analyses identified independently active brain regions exhibiting functional connectivity. The Kolmogorov Smirnov test was used to assess pairwise effects in localized voxel activation in the Fourier domain, and Kendall’s τ was used to assess functional connectivity among seed regions. *P*-values for rs-fMRI were corrected to account for the false discovery rate (FDR) due to multiple testing [52]. Data were log-transformed to ensure normality of distribution and analyzed using SPSS (SPSS Inc, Chicago, IL) or AFNI utility 3dMEMA, respectively. All values reported here are means ± standard error (SE).

### Data and resource availability

Data generated and/or analyzed in the current study are available from the corresponding author on reasonable request.

## Results

### Acute central insulin or IGF-1 can restore whole-body insulin action to more youthful levels

**Fig. 1:**
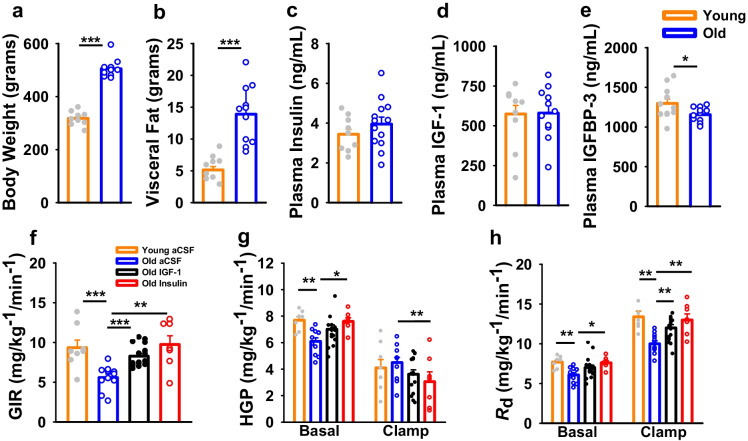
Acute central insulin or IGF-1 can restore whole-body insulin action in old rats to more youthful levels **a** old rats have greater total body weight compared with young rats, as well as **b** increased VF. **c–d** Approximately 4-h fasting plasma insulin and IGF-1 levels were not significantly different between young (*n* = 10) and old rats (*n* = 11), although **e** plasma IGFBP-3 levels were reduced with age. **f** Hyperinsulinemic-euglycemic clamps were performed with concomitant ICV infusion with aCSF (young *n* = 8; old *n* = 11), old IGF-1 (*n* = 16), or old insulin (*n* = 8). The glucose infusion rate (GIR) required to maintain euglycemia was reduced in old aCSF rats compared with young controls whereas both insulin and IGF-1 infusion in old rats restored GIR to young levels. **g–h** Suppression of hepatic glucose production (HGP) under hyperinsulinemia did not differ with age, but the ability of insulin to stimulate glucose disposal rates (*R*d) was substantially lower in old animals. However, ICV insulin was able to both enhance suppression of HGP, and increase glucose disposal, relative to old control animals, was likewise lower under basal conditions in old relative to young aCSF rats but was partially restored in IGF-1 and insulin rats; under clamped conditions, old IGF-1 and old insulin rats displayed lower HGP than young aCSF animals. **h** Glucose disposal rate (*R*_d_) was lower in old compared with young rats but was restored to levels comparable to young aCSF rats in old IGF-1 and old insulin rats under basal conditions; old IGF-1 levels were intermediate with clamp, whereas old insulin *R*_d_ was comparable to that observed in young aCSF rats. Young versus old comparisons were conducted using an independent sample *t*-test while clamp data were analyzed via one-way ANOVA. Vertical bars and lines represent mean ± SE. Different letters indicate significant differences between groups determined by Tukey honest significant differences (HSD) *p ≤ 0.05, **p ≤ 0.01, ***p ≤ 0.001

Basic phenotypic characteristics were first assessed in younger and older male FBN rats, which confirmed that old animals were heavier and had a three-fold increase in visceral fat mass, relative to young (Fig. 1a–b; *p* < 0.001). Surprisingly, no differences were observed in fasting plasma insulin or IGF-1 levels between young and old animals, but circulating IGFBP-3 levels were reduced with age (Fig. 1c–e; *p* < 0.05). Next, we performed hyperinsulinemic-euglycemic clamp studies with concomitant ICV infusion of vehicle, IGF-1 or insulin. Relative to young animals, the GIR required to maintain euglycemia under hyperinsulinemic conditions was ~ 50% lower in old rats (Fig. 1f; *p* < 0.001). This effect was not due to an inability to suppress HGP (Fig. 1g), but rather from markedly impaired glucose disposal (Fig. 1h; *p* < 0.001). Despite older animals demonstrating visceral obesity and whole-body insulin resistance, acute ICV infusion of either IGF-1 or insulin was able to increase the GIR toward more youthful levels, which in the case of ICV insulin was due to both enhanced suppression of HGP and glucose disposal, relative to old control animals (Fig. 1f–h; *p* < 0.01).

### *Manifestation of brain aging indicators in old male FBN rats *via* measured declines in cognitive function, synaptic markers, and increased expression of neuroinflammatory genes*

**Fig. 2:**
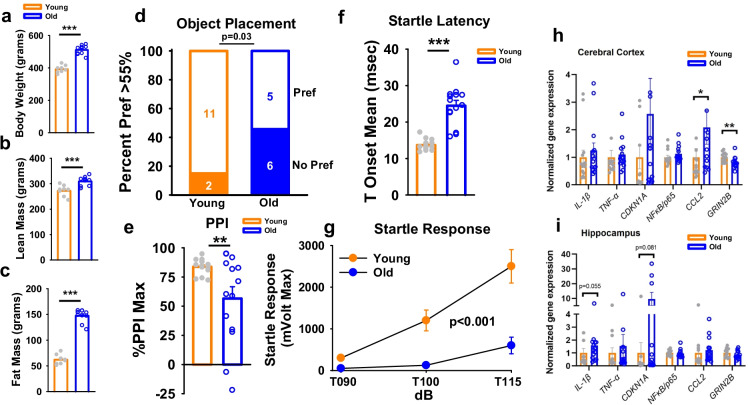
Old male FBN rats demonstrated many features of cognitive decline and increased markers of neuroinflammation. In old rats, **a** body weight, **b** lean mass, and **c** fat mass were significantly greater than in young rats (*n* = 10 per group). **d** Preference for novel object placement in the object placement (OP) test in young (*n* = 13) and old (*n* = 11) rats. At a discrimination threshold of 55%, significantly fewer old rats relative to young rats preferred the novel object. **f–g** Old rats exhibited deficits in acoustic startle response, with significant decrements in prepulse inhibition (PPI, as determined by %PPI Max), twofold increased startle latency (defined as the time to the onset of the startle response, T Onset mean), and reduced displacement of a piezoelectric platform in response to an auditory stimulus at three volumes (90, 100, and 115 dB; *p* ≤ 0.001). **h–i** RT-qPCR was performed to assess age-related changes in expression of selected genes in cerebral cortex and hippocampus in Young (*n* = 13) and old (*n* = 14) rats. Young versus old comparisons were conducted using an independent sample *t*-test while OP preference (d) was analyzed via the chi-square test. Bar and line graphs represent mean ± SE. Expression was normalized to that of *Actb* and quantified as fold-change in old relative to young using the ΔΔCt method. *p ≤ 0.05, **p ≤ 0.01, ***p ≤ 0.001

We next aimed to determine if evidence of central insulin resistance might exist in other contexts. To this end, a separate cohort of old and young FBN rats (*n* = 14 per group) were initially phenotyped and then subjected to a battery of behavioral assays to confirm the manifestation of age-related changes in cognition in this strain. Consistent with rats assigned to clamp studies, old animals were heavier and harbored greater lean and fat mass (Fig. 2a–c; *p* < 0.001). Consistent with many prior observations in aged male FBN rats [53], aged animals had confirmed cognitive deficits, since when subjected to a novel OP test, less than half of old animals preferred the novel object, as compared to ~ 85% of young animals (Fig. 2d; *p* < 0.05). As a somewhat more novel observation, age-associated deficits were also observed in the acoustic startle response, with reduced maximum pre-pulse inhibition (%PPI max; Fig. 2e; *p* < 0.01), increased startle latency (T onset mean), and diminished startle response at 90–110 dB volume (*p* < 0.001 for trend) in old rats (Fig. 2f–g), which importantly were not due to hearing deficits per se. Moreover, inflammatory and synaptic markers were assessed in cortical and hippocampal tissues via qPCR. Indeed, increased age-related expression of the chemotactic ligand *ccl2* was observed in old cortex (Fig. 2h; *p* < 0.05), while a trend toward increased *cdkn1a* (*p* = 0.08) and the inflammatory cytokine *il1β* (*p* = 0.055) was observed in hippocampus with age (Fig. 2i). Moreover, the expression of the NMDA receptor subtype 2b, *grin2b*, was significantly reduced in cortex (*p* < 0.01), but not in hippocampus.

### Resting state-BOLD fMRI reveals reduced neural connectivity and hippocampal complexity with age

**Fig. 3:**
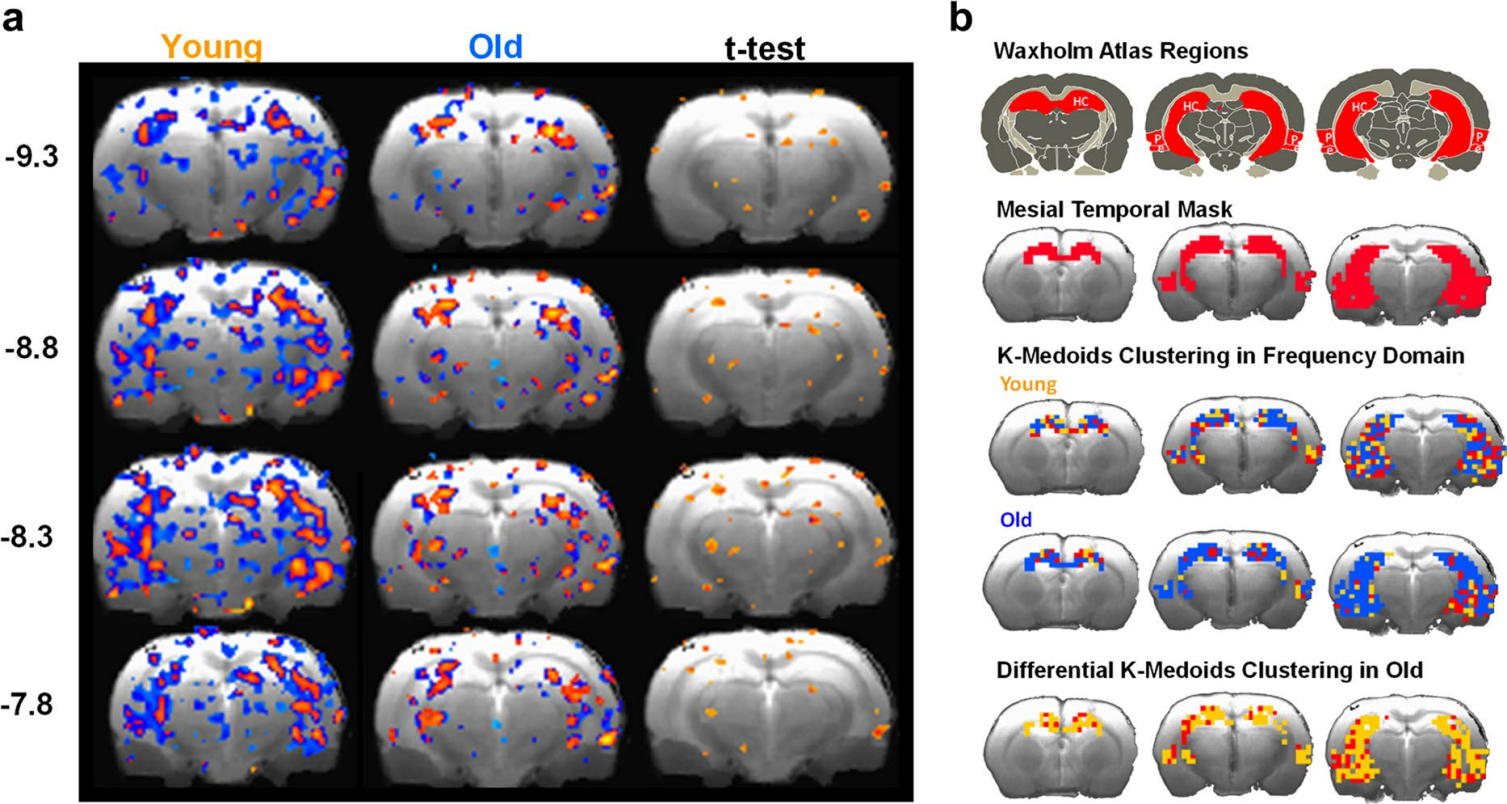
Reduced neural connectivity and complexity in the aged rat hippocampus **a** functional connectivity was performed with the mesial temporal (MT) region as the “seed” and connectivity to other brain regions were assessed in young (*n* = 8) and old rats (*n* = 9). In aging, a significant loss of connectivity was observed between the MT and entorhinal cortex, thalamus, and dentate gyrus where indicated by independent sample *t*-tests with FDR correction (*p* < 0.05) **b** K-medoids analysis in mesial temporal region (Waxholm atlas region shown with mesial temporal mask) reveals differential k-medoids clustering in aged relative to young rats. More heterogeneous mesial temporal clustering in the frequency domain is also observed in young rats

To determine whether manifestations in cognitive aging could be further substantiated in vivo, we next performed rs-BOLD fMRI in young and old FBN male rats to determine if decrements in neural network connectivity could be detected. Functional connectivity was performed with the mesial temporal (MT) region as the “seed” and connectivity to other brain regions was assessed. In aging, a significant loss of connectivity was observed between the MT and entorhinal cortex, thalamus, and dentate gyrus (Fig. 3a; *p* < 0.05 for highlighted regions via FDR adjusted *t*-test). K-medoids clustering in the frequency domain was next performed. To this end, a MT mask was applied using Waxholm brain atlas regions and K-medoids clustering in MT was evaluated in young and old rats. Interestingly, these data revealed that clustering heterogeneity in the frequency domain was reduced in old rats, indicating a loss of signal complexity in this region, suggesting a reduced information density in BOLD signal fluctuations in the aged rat brain (Fig. 3b; *p* < 0.05 for old vs young; FWER corrected at cluster level).

### The brain region-specific BOLD signal responds to ICV insulin in old, but not young rats

**Fig. 4:**
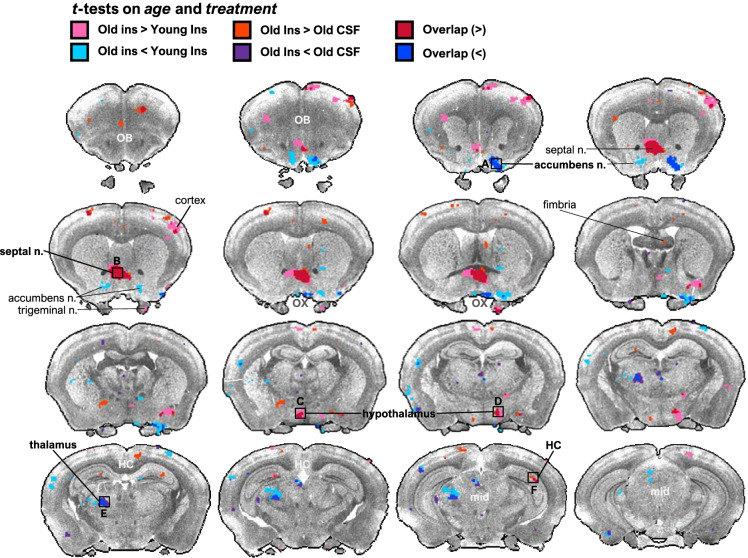
Central insulin elicits distinct brain regional effects on the rs-fMRI signal in old, but not young rats. Young (*n* = 8) and old rats (*n* = 9) were assigned to a crossover design whereby animals were randomly assigned to either ICV CSF or insulin first and then received the other treatment 1 week later. The rs-fMRI imaging session was 1 h in length and BOLD signal intensity was measured in extracted ROIs. No differences were observed in ΔBOLD signal between young and old rats treated with CSF. However, ICV insulin surprisingly failed to significantly alter the BOLD signal in any region in young rats but led to several dynamic changes in BOLD signal across the brain in old. Specifically, ICV-insulin treated animals exhibited significantly increased mean BOLD signal intensity in septal nucleus, hypothalamus, and hippocampus and decreased intensity in thalamus and nucleus accumbens and compared to old CSF and young insulin treated animals. Highlighted regions indicat significantly different BOLD signal intensity between age (independent *t*-test on ΔBOLD signal, treatment (paired *t*-test within age group), or both determined; where pink, aged insulin > young insulin; orange, aged insulin > aged aCSF; red, aged insulin > young insulin and aged CSF; turquoise, aged insulin < young insulin; indigo, aged insulin < aged aCSF; blue, aged insulin < young insulin and aged aCSF. Regions of interest (ROIs) were identified and projected in Waxholm space

Given that aged rats had several confirmed manifestations of aging, including whole-body insulin resistance, as well as cognitive and neural deficits, we were next interested in whether evidence of brain insulin resistance could be identified in old rats via an impaired ability of the BOLD signal to respond to ICV insulin in the aged brain. Young and old rats were assigned to a crossover design whereby animals were randomly assigned to either ICV CSF or insulin first and then received the other treatment 1 week later. The rs-fMRI imaging session was 1 h in length and BOLD signal intensity was measured in extracted ROIs. No differences were observed in ΔBOLD signal between young and old rats treated with CSF. However, ICV insulin surprisingly failed to significantly alter the BOLD signal in any region in young rats but led to several dynamic changes in BOLD signal across the brain in old. Specifically, ICV-insulin-treated animals exhibited significantly increased mean BOLD signal intensity in septal nucleus, hypothalamus, and hippocampus, and decreased intensity in thalamus and nucleus accumbens and compared to old CSF and young insulin-treated animals (Fig. 4).

### *Insulin-stimulated Akt phosphorylation *ex vivo* is impaired in old rat brain*

**Fig. 5:**
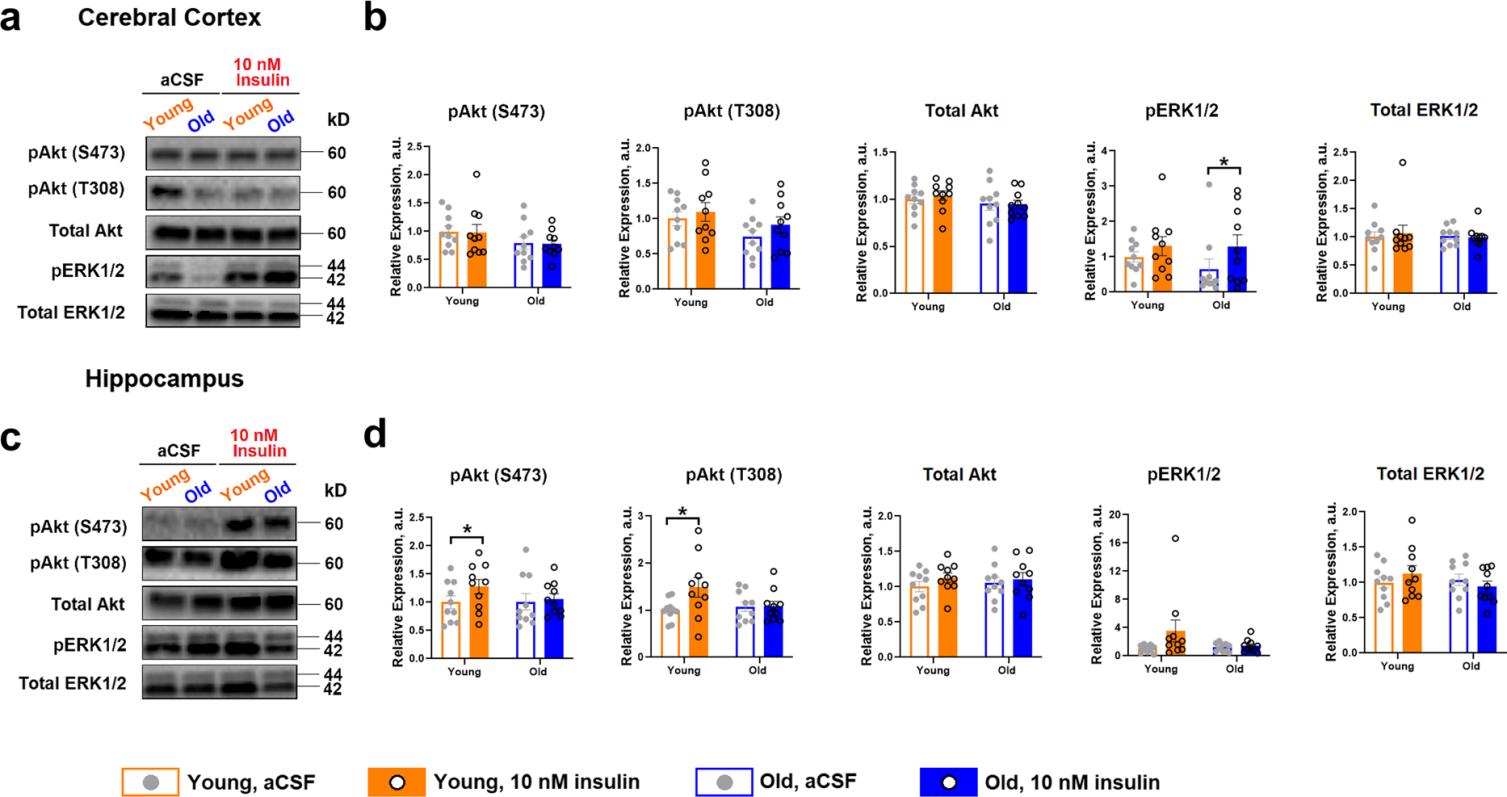
Ex vivo insulin exposure in rat cerebral cortex and hippocampus and modulation of PI3K/Akt and MAPK signaling pathways. Approximately 20 mg of cerebral cortex and hippocampus was each collected from young and old rats immediately after sacrifice and incubated for 2 min either in aCSF or 10 nM insulin in a 37 °C incubator, 5% CO_2_ (*n* = 10 per group) following 10-min untreated thermal and biological equilibration (12-min total incubation time). **a–b** Tissue responsiveness to 10 nM insulin in cortex, indicated by phosphorylation of Akt on Ser473 or Thr308 residues indicated no effect in altering either pAkt S473 or T308 phosphorylation. However, insulin tended to increase pErk levels in cortex, but this was only significant in old animals. **c–d** In hippocampus, insulin was able to significantly activate pAkt in young, as evidenced by increased phosphorylation at S473 and T308, but not in old. There were also no significant effects detected in the Erk pathway, collectively suggesting discordance regarding insulin effects with age among in vivo and ex vivo assays. Data were analyzed as a paired *t*-test within each age group. Representative Western blots are shown. Bar graphs represent mean ± SE. *p ≤ 0.05

Based upon evidence of preserved or even heightened CNS insulin responses with age in the context of hyperinsulinemic clamps and fMRI, respectively, we next assessed the ability of insulin to activate canonical signaling directly in cortical and hippocampal tissue ex vivo. In cortex, 10 nM insulin for 2 min failed to significantly alter either pAkt S473 or T308 phosphorylation. However, insulin tended to increase pErk levels in cortex, but this was only significant in old animals (Fig. 5a–b; *p* < 0.05). However, in hippocampus, insulin was able to significantly activate pAkt, as evidenced by increased phosphorylation at S473 and T308 (Fig. 5a–b; *p* < 0.05), but no significant effects were detected in the Erk pathway, collectively suggesting discordance regarding insulin effects with age among in vivo and ex vivo assays.

### Insulin and IGF-1 ligands, but not receptors, decline in the aged FBN rat brain

**Fig. 6:**
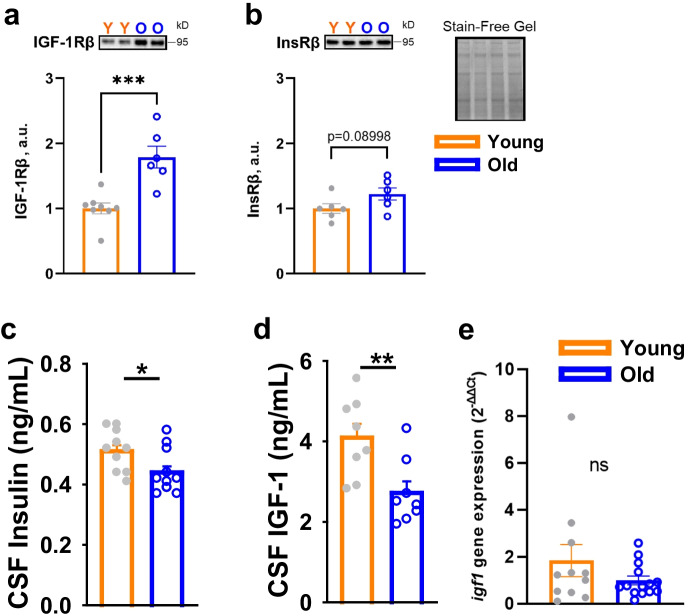
Reduced CSF insulin and IGF-1 concentrations suggest impaired input from the periphery in aged rats. **a–b** IGF-1R levels were markedly increased in the old hippocampi from old rats, relative to young rats, while InsR levels also tended to be elevated with age. **c–d** CSF concentrations of insulin and IGF-1 were significantly lower in old animals (*n* = 10 per group). **e** Relative expression of *igf1* mRNA in young (*n* = 11) and old (*n* = 14) rat hippocampus revealed no significant difference in transcript level with age. Young versus old comparisons were conducted using an independent sample *t*-test. Bar graphs represent mean ± SE. *p ≤ 0.05, **p ≤ 0.01. ***p ≤ 0.005

In order to gain a more comprehensive understanding regarding the disparate ability for insulin to activate canonical signaling, we more carefully examined the effect of age on relevant receptor and ligand levels in the CNS. Surprisingly, we observed that IGF-1R levels were markedly increased in old hippocampus, relative to young (Fig. 6a; *p* < 0.001), while InsR levels also tended to be elevated with age (Fig. 6b; *p* = 0.08998). However, these changes coincided with significantly lower CSF concentrations of insulin (Fig. 6c; *p* < 0.05) and IGF-1 (Fig. 6d; *p* < 0.01), suggesting that CNS input of these ligands might be attenuated, which was supported in part by unchanged *igf1* mRNA expression with age in hippocampus (Fig. 6e).

## Discussion

There has been a great deal of attention focused on the concept of brain insulin resistance, a phenomenon proposed to link T2D and metabolic dysfunction with age-related cognitive decline [24, 54]. Indeed, there is evidence that high-fat diet and obesity [55], T2D [56], AD [24], and stress [57] can provoke an insulin resistant–like state in the CNS. Moreover, a myriad of reports has observed impaired canonical insulin signaling in rodent AD models as well as post-mortem human AD brains [24, 54]. However, the assertion that brain insulin resistance is a salient feature of aging, T2D and dementias require a careful evaluation of whether brain insulin resistance is identical at the molecular and physiological level to insulin resistance as traditionally defined in peripheral tissues. Thus, a greater understanding of how insulin enters the brain [58, 59], where and how it acts, and what features best predict its (in)ability to signal is important. However, defining insulin resistance in the brain has proven difficult given its privileged, tightly-regulated access, distinct neuroanatomical regions, unique electrophysiological properties, and a heterogenous composition of neurons that comprise a highly complex collection of circuits along with several supporting cells [14]. As such, canonical signaling responses in bulk tissue, rather than discrete effects on defined responses, regions, cell types, and/or circuits are likely inadequate to fully capture brain insulin resistance. Indeed, our study observed substantial discordance when comparing insulin responses via functional assays in vivo versus signaling ex vivo, as demonstrated here by evidence that insulin responsiveness in the aging rat brain appears to be intact and/or even enhanced, in spite of a diminution in Akt activation by insulin in aged brain tissue.

By all accounts, old FBN rats used in this study demonstrated ample evidence of age-related decrements, including whole-body insulin resistance, cognitive deficits, and diminished resting-state network coherence relative to young (4–5 months) rats. We also characterized age-related changes in CNS gene expression of chemotactic, inflammatory, and senescent markers. *Grin2b* mRNA, which encodes glutamate (NMDA) receptor subunit ε2, was significantly decreased in cerebral cortex of aged rats. A broad array of synaptic markers, including GRIN2A and GRIN2B, is often downregulated in prefrontal cortex in advanced age [60] and in AD [61], with loss of components of the excitatory synapse, thereby potentially contributing to the modest cognitive deficits seen here in old animals. *Ccl2*, which encodes for monocyte chemoattractant protein-1 (MCP-1), showed a greater than two-fold elevation in aged rat cortex, and in other studies has been linked to AD-associated cognitive decline [62], tauopathy [63], and age-related BBB endothelial cell dysfunction [64]. Moreover, rs-fMRI and seed-based analysis revealed a significant loss in connectivity between the entorhinal cortices, a region important to memory formation, and other brain regions in old animals. Aging was also found to associate with a decrease in signal heterogeneity via K-medoids analysis in entorhinal cortices and the CA3 hippocampal field.

These imaging-based observations were accompanied by evidence from OP and acoustic startle response tests, respectively, confirming cognitive deficits exist in this strain, as has been shown prior [53], demonstrating diminished preference for novel objects, as well as increased startle latency, reduced startle response, and decreased PPI at 40-ms inter-stimulus intervals in old rats, which were not due to hearing deficits per se. Decrements in PPI have also been demonstrated in some studies of human patients [65] and in rodent models of AD (66–68) and aging (67, 69–72). Collectively, these findings suggest a reduced information density in BOLD signal fluctuations in the aged rat brain and together reveal a diffuse loss of sub-regional network integrity that is associated with cognitive and neural deficits. Such effects are consistent with an elegant study from Ash [66], in which the authors demonstrated via rs-fMRI that the greatest loss in functional connectivity in aged rats corresponded to those with impaired memory. Interestingly, some aged rats in that study had preserved memory, and those animals demonstrated a network signature that was distinct from both young and old-cognitive impaired animals, perhaps representing a compensatory attempt by the aged brain to maintain memory [66].

An interesting observation in this study was the relatively intact insulin responsiveness with age, including a heightened sensitivity to insulin in driving deviations in BOLD signal for old, but not young rats undergoing fMRI scans. This was demonstrated by a significant enhancement in BOLD signal intensity in areas of the septal nucleus, hypothalamus, and hippocampus and a reduction in BOLD signal in discrete regions of the nucleus accumbens and thalamus. However, it is not feasible to infer functionality from the direction of BOLD signal change, given the complexity of neural circuits and indirect effects which may have a net stimulatory or inhibitory effect on specific neuronal populations. Thus, we conclude that old rats demonstrate a heightened response to acute central insulin exposure in this context. Likewise, despite impaired insulin sensitivity under clamp conditions in old versus young controls, central administration of insulin or IGF-1 were both effective at restoring GIR, a measure of whole-body insulin action, to more youthful levels. This observation is generally in agreement with prior work from our lab where aged Sprague–Dawley rats centrally treated with insulin via ICV delivery demonstrated partial rescue of whole-body insulin action, though these effects were more potent with IGF-1 treatment, perhaps owing to greater evidence of somatopause in this strain [67].

While the notion that insulin responsiveness may be maintained or even heightened in the aged or diseased brain seems counterintuitive, it is important to note that similar observations have been noted by others [68], including an enhanced ability of insulin to trigger calcium fluxes in hippocampal slices from older animals and promote cerebral blood flow in the aged rat brain [69, 70]. Likewise, insulin administered directly to the hippocampus, but not given peripherally during a hyperinsulinemic clamp, was able to increase insulin signaling in younger and older *APP/PSEN* mice [71]. Moreover, we found that CSF levels of insulin and IGF-1 were significantly reduced in old rats, relative to young, despite comparable levels in the periphery. Similar observations have been reported for CSF insulin in aging and T2D, though the extent to which a reduction in CSF insulin levels serves as a meaningful surrogate of lower insulin in the brain interstitial fluid (BISF) is unclear [58]. Indeed, impaired insulin (and IGF-1) input into the CNS has been proposed as a mechanism of their reduced action in the brain by others [72–75], and our data would support the notion that this “insulin starved” scenario may indeed contribute to reduced brain insulin action with age.

These reductions in growth factors coincided with greater IGF-1R content and a trend toward higher InsR levels detected in the aged FBN rat hippocampus. However, in other strains, such as SD rats, the effect of age on IGF-1R expression has been less clear, with contrasting reports of increased IGF-1Rs in the CA3 hippocampal field in 24–29 month animals [76], diffusely downregulated IGF-1R in 22-month animals of the same strain [77], while we previously observed no overall difference in IGF-1R levels with age in SD hypothalamus, though greater variability was observed in older animals [10]. Thus, age-related levels in brain IGF-1Rs do not appear to undergo a generalized change with aging and seem to require a more granular analysis that account for a number of variables, including species, strain, sex, brain region, and possible age-related shifts in cellular composition of the parenchyma.

In contrast to brain insulin, which is derived mostly if not exclusively from pancreatic β-cell secretion, IGF-1 in the CNS is verifiably attributable to both peripheral input and local production [50]. Liver is the major source of endocrine IGF-1 and circulates as part of a ternary complex consisting of IGFBPs and acid labile subunit (ALS), of which IGFBP-3 comprises > 80% of circulating IGFBPs and has been used as a marker for IGF-1 bioavailability [78]. We observed a slight but significant decrease in plasma IGFBP-3 with age, despite no change in plasma IGF-1. Although not directly measured here, IGFBP-1 and IGFBP-2 are also often reduced in aging and insulin-resistant states [79, 80]. Given IGF-1 levels were static with age in this strain, one might predict that the pool of free circulating IGF-1 was increased in old animals, thereby further implicating a potentially impaired BBB transport of IGF-1 in aging, as has been previously proposed. On the other hand, neurons and glial cells can also synthesize IGF-1 locally, and a decline here could also contribute to lower IGF-1 in the CNS. However, in agreement with previous findings [81], we observed no age-related change in *igf1* gene expression in hippocampus. Although speculative, enhanced proteolytic degradation of growth factors in the aged or diseased CNS is another possibility of lower levels in the brain that cannot be ruled out [82], though this would need to be directly tested.

An important limitation of this study is that because only males were studied, we cannot adequately address here the role of sex differences as it relates to CNS responsiveness in the aged brain. Indeed, sex as a biological variable in the insulin/IGF-1 pathway and aging is well established. Moreover, several reports have suggested unique benefits to restorative levels of insulin and related growth factors in the brain, and we have previously found that at least as it relates to IGF-1, such strategies preferentially benefit male mice [50]. To this end, the extent to which brain insulin and/or IGF-1 responsiveness is preserved or lost in females with aging is a critical question that should be more carefully examined, as it could hold implications for why AD and cognitive decline are generally more prevalent in women. Such efforts that also incorporate a CNS-targeted treatment arm for effects on functional connectivity and related behavioral outcomes in both sexes are also a fruitful area for future investigation.

In summary, brain insulin resistance remains conceptually important in its implications, but quite elusive in its understanding. Similar to the periphery, oxidative stress, mitochondrial dysfunction, lipotoxicity, and inflammation have all been linked to impaired CNS insulin action by impeding canonical signaling [83]. Likewise, proteopathies, such as β-amyloidosis and tauopathy in AD, also purportedly induce an insulin-resistant–like state [24]. However, these and other data also suggest that the impaired CNS input of these ligands may also comprise a very important facet of cerebral hypometabolism and diminished brain insulin action, supporting the notion that central insulin and IGF-1 replacement strategies that bypass the BBB, such as intranasal treatment, could hold therapeutic potential for the aged CNS and related diseases. Moreover, given the sheer complexity and diversity of the brain, assays that rely on detecting generalized PI3K-Akt activation and other downstream effectors in bulk tissue may lack the specificity necessary to draw definitive conclusions with regard to its functional effects. Indeed, our data highlight the complexity of brain insulin action by demonstrating disparate responses to insulin in canonical signaling versus imaging-based and integrated in vivo metabolic outcomes. Thus, studies aiming to gain a more sophisticated and incisive understanding of how aging, sex differences, and metabolic and degenerative diseases do and do not perturb the salutary functions of the CNS insulin, and related factors are warranted.
